# Mechanisms of combined deer antler polysaccharides and postbiotics supplementation for regulating obesity in mice

**DOI:** 10.29219/fnr.v69.11634

**Published:** 2025-01-28

**Authors:** Lanyue Yin, Jiating Li, Xueyue Tai, Guoqi Zhang, Mingran Luan, Bao Zhong, Fenglin Li

**Affiliations:** 1 College of Food Science and Nutrition Engineering, Jilin Agricultural Science and Technology University, Jilin, China; 2 School of Public health, Jilin Medical University, Jilin, P.R. China; 3 College of Food Science and Engineering, Changchun University, Changchun, China

**Keywords:** deer antler polysaccharides, postbiotics, lipid metabolism, regulatory effects

## Abstract

**Objective:**

This study investigated the mechanisms related to lipid metabolism regulation after combined supplementation with deer antler polysaccharides and postbiotics.

**Methods:**

Thirty-two male mice were divided into high-fat diet, HD + deer antler polysaccharides, HD + *Bacillus coagulans* postbiotics, and HD + deer antler polysaccharides + *B. coagulans* postbiotics groups. The diets contained 60% fat. After 9 weeks, the effects of deer antler polysaccharides and postbiotics on lipid metabolism were assessed through blood biochemical, histological tissue staining, and polymerase chain reaction analyses.

**Results:**

Supplementation with deer antler polysaccharides and postbiotics significantly inhibited weight gain in obese mice, reduced serum total cholesterol, triglyceride, and low-density lipoprotein levels and markedly increased the serum high-density lipoprotein level. Additionally, hepatic lipid droplet accumulation and adipocyte hypertrophy improved. The expressions of the lipid synthesis genes, sterol regulatory element-binding protein 1 (i.e. *SREBP-1c*), and fatty acid synthase (i.e. *FAS*), significantly decreased, while peroxisome proliferator-activated receptor alpha (i.e. *PPAR-α*) and acyl-CoA oxidase 1 (i.e. *ACOX1*) expression significantly increased. The expressions of the inflammation-related genes, tumor necrosis factor-alpha (i.e. *TNF-α*), interleukin (*IL*)*-6*, and *IL-1* also significantly decreased.

**Conclusion:**

Thus, combined deer antler polysaccharides and postbiotic supplementation regulated obesity in mice, potentially by modulating lipid synthesis and inflammation-related gene expression.

## Popular scientific summary

- The combined use of antler polysaccharides and postbiotics has a significant effect on lipid regulation.
- Supplementation of antler polysaccharides and postbiotics can significantly inhibit the weight gain of obese mice, reduce serum total cholesterol, triglyceride and low-density lipoprotein levels, and significantly increase serum high-density lipoprotein levels.
- Supplementation of antler polysaccharides and postbiotics can improve liver lipid droplet accumulation and adipocyte hypertrophy.
- Supplementation of antler polysaccharides and postbiotics can regulate the expression of lipid synthesis genes sterol regulatory element binding protein 1, fatty acid synthase peroxisome proliferator-activated receptor α and acyl-CoA oxidase 1.
- Supplementation of antler polysaccharides and postbiotics can regulate the expression of inflammation-related genes tumor necrosis factor-α, interleukin-6, and interleukin-1.

Obesity is a growing global health concern and a complex metabolic disorder characterized by excessive fat accumulation. Obesity substantially elevates the risk of developing several health conditions, such as cardiovascular diseases, type 2 diabetes, and certain types of cancer (1). The global burden of obesity has reached alarming levels, with recent studies indicating that approximately 39% of adults worldwide are overweight or obese (2). Obesity is primarily the result of an imbalance between energy intake and expenditure, often influenced by genetic, environmental, and behavioral factors. Diet, physical activity, and genetic predisposition play crucial roles in its development (3). Excessive calorie consumption, particularly of high-fat and high-sugar diets, coupled with a sedentary lifestyle, are key contributors to the obesity epidemic (4). In addition to the direct impact on physical health, obesity is also associated with alterations in endocrine functions, such as dysregulated adipokine production and insulin resistance (5). At the molecular level, obesity is characterized by altered lipid metabolism, chronic low-grade inflammation, and dysfunction in several key signaling pathways, including those involving insulin, leptin, and adiponectin. These disruptions contribute to a vicious cycle of metabolic dysregulation and inflammatory responses, further exacerbating obesity and its associated comorbidities (6, 7). Recent studies have focused on the molecular mechanisms underlying obesity, particularly the role of key regulatory genes (8, 9). Sterol regulatory element-binding protein 1c (SREBP-1c) and fatty acid synthase (FAS) enzymes are pivotal in lipogenesis, thus promoting fat storage and synthesis (10, 11). Conversely, peroxisome proliferator-activated receptor alpha (PPAR-α) and acyl-CoA oxidase 1 (ACOX1) play critical roles in fatty acid oxidation and energy expenditure (12, 13). Additionally, obesity is associated with a state of chronic low-grade inflammation, wherein pro-inflammatory cytokines, such as tumor necrosis factor-alpha (TNF-α), interleukin-6 (IL-6), and interleukin-1 (IL-1), are upregulated, further exacerbating metabolic dysregulation (14–16). Understanding these interactions is crucial for developing effective therapeutic strategies against obesity. Current strategies for managing obesity primarily involve lifestyle interventions, including dietary modifications and increased physical activity. However, these approaches are often challenging to implement and maintain in the long term (17). Pharmacological and surgical interventions are also available but are typically associated with various side effects and risks (18). As a result, there has been increasing interest in exploring alternative therapeutic strategies, including natural products and postbiotics, which may provide a safer and more sustainable option for obesity management (19, 20).

Deer antler velvet is a traditional Chinese medicinal product derived from the antlers of deer (21). It has been utilized in various cultures, especially in East Asia, for centuries due to its purported health benefits. In recent years, scientific interest in deer antler velvet has grown, with studies investigating its bioactive components and their potential therapeutic applications in various physiological and pathological conditions, including obesity, inflammation, and immune dysfunction (22, 23). It contains various bioactive compounds, including polysaccharides, amino acids, and minerals (24). Consequently, it has been used for its purported health benefits, including anti-inflammatory and anti-obesity effects. One of the most well-documented effects of deer antler velvet is its anti-inflammatory properties. Studies have shown that DAV supplementation can reduce the levels of pro-inflammatory cytokines such as TNF-α, IL-1, and IL-6, which are involved in the pathogenesis of various chronic diseases, including obesity (25, 26). This anti-inflammatory effect is thought to be mediated by the modulation of the NF-κB signaling pathway, a key regulator of inflammation (27). Research has also highlighted the potential of deer antler velvet in regulating lipid metabolism. Several studies have reported that DAV supplementation can decrease total cholesterol (TC), triglycerides (TG), and low-density lipoprotein cholesterol (LDL-C), while increasing high-density lipoprotein cholesterol (HDL-C), suggesting its beneficial role in managing dyslipidemia and preventing cardiovascular diseases (28). The underlying mechanisms may involve the modulation of key lipid metabolism-related enzymes and the improvement of lipid transport and storage in adipocytes. Recent studies have highlighted the potential of deer antler polysaccharides (DAPs) to modulate lipid metabolism and reduce body weight in animal models. DAPs have been shown to inhibit the expression of lipogenic genes, such as *SREBP-1c* and *FAS*, while promoting the expression of fatty acid oxidation-related genes, like *PPAR-α* and *ACOX1* (29). Furthermore, DAPs exhibit anti-inflammatory properties, which help reduce pro-inflammatory cytokines associated with obesity, making them a promising candidate for obesity management.

Postbiotics have also emerged as a novel approach to modulating metabolic health (30). Recent studies have demonstrated that postbiotics can improve gut microbiota composition, enhance gut barrier function, and regulate inflammatory responses, thereby benefitting obesity (31). Postbiotic administration has also been linked to improved insulin sensitivity and reduced body weight, emphasizing their role in the prevention and treatment of obesity-related metabolic disorders (32). *Bacillus coagulans* has been extensively studied for its spore-forming ability and lactic acid production capacity, and its probiotic characteristics have also been widely investigated. *B. coagulans* produces valuable metabolites, examples include lactic acid and polysaccharides, which are recognized for their health benefits, such as anti-inflammatory properties, immune system modulation, and regulation of lipid metabolism. Recent studies have highlighted the potential of *B. coagulans* postbiotics for managing obesity (30). *B. coagulans* postbiotics can downregulate lipogenic genes, such as *SREBP-1c* and *FAS*, which are involved in fat storage, while upregulating genes, like *PPAR-α* and *ACOX1*, that promote fatty acid oxidation. This shift in gene expression helps reduce fat accumulation and enhance energy expenditure (33). Postbiotics derived from *B. coagulans* have been demonstrated to reduce the levels of pro-inflammatory cytokines, such as TNF-α, IL-6, and IL-1. Therefore, these postbiotics improve metabolic health by reducing chronic inflammation, significantly contributing to obesity-related complications (34).

The significance of combining DAPs with postbiotics lies in their complementary mechanisms of action. While DAPs directly influence lipid metabolism and inflammatory pathways, postbiotics enhance gut health and modulate systemic metabolic responses. This combination could synergistically inhibit weight gain, improve serum lipid profiles, and reduce inflammation in obesity models. By elucidating the mechanisms by which DAPs and postbiotics exert their effects, we aim to provide insights into potential therapeutic interventions for obesity. This study helps clarify the interplay between natural compounds and metabolic regulation and highlights the importance of integrating traditional medicine with modern nutritional science in the fight against obesity.

## Materials and methods

### DAPs preparation

Sika deer antler powder was mixed with water at a 1:10 ratio, and the solid residue was removed by filtration using 40 kHz ultrasound for 30 min. The filtrate was then concentrated under reduced pressure using a rotary evaporator to obtain a more concentrated polysaccharide solution. Subsequently, 70% (v/v) ethanol was introduced, and the mixture was kept at 4°C overnight. The resulting polysaccharide precipitate was collected through centrifugation, followed by freeze-drying to yield a stable powder suitable for long-term storage and subsequent analysis or application. The purity and content of DAPs were determined by phenol-sulfuric acid method.

### Preparation of B. coagulans MZY531 postbiotics

The *B. coagulans* MZY531 strain (Jilin Mingzhiyuan Biotechnology Co. Ltd., Changchun, China) was cultured on a Luria-Bertani agar plate, then transferred into glucose yeast extract peptone liquid medium and incubated at 50°C with shaking at 1,800 rpm for 24 h. Following centrifugation (3,000 rpm, 4°C, 10 min), the bacterial pellet was collected and resuspended in a sterile isotonic sodium chloride solution, adjusting the bacterial concentration to 1.0 × 10^9^ CFU/mL. A 50 mL aliquot of this *B. coagulans* MZY531 suspension was subjected to ultrasonic disruption in an ice bath for 15 min at 800 W using an ultrasonic processor. The resulting liquid was then vacuum freeze-dried to obtain a concentrated post-biotic powder of *B. coagulans MZY531.*

### Animal model establishment

Thirty-two male ICR mice (Yisi Experimental Animal Technology Co., Ltd., Changchun, China) were housed in a controlled environment at 23 ± 2°C with 55 ± 5% relative humidity under a 12-h light/dark cycle for 1 week. After acclimatization, the mice were randomly assigned to four groups: high-fat diet (HD), high-fat diet with deer antler polysaccharides (HDAPs), high-fat diet with *B. coagulans* postbiotics (HBCP), and high-fat diet with both deer antler polysaccharides and *B. coagulans* postbiotics (HDBP). The diet consisted of 60% fat. During the study, the HD group administered water via gavage. After 9 weeks, the mice were fasted for 12 h before euthanasia. Blood was collected, centrifuged at 10,000 rpm for 5 min at 4°C to isolate serum, and liver and epididymal fat tissues were harvested. All samples were stored at –80°C for subsequent analysis.

### Determination of biological parameters in serum

Total cholesterol (TC), triglyceride (TG), LDL-C, and HDL-C levels in the serum were measured using kits following the manufacturer’s instructions (Jiancheng Bioengineering Institute, Nanjing, China).

### Histological section staining

Liver tissue and abdominal fat were preserved overnight in 10% formalin solution, then embedded in paraffin, and cut into 4-μm thick sections. The sections were deparaffinized using xylene and ethanol and washed with deionized water for 5 min. Staining was performed using hematoxylin and eosin, and the sections were photographed using a microscope at 200 × magnification.

### Real-time fluorescent quantitative polymerase chain reaction for measuring liver lipogenesis and inflammation-related gene expression

Initially, 50 mg of liver tissue was homogenized in 500 μL of TRIzol using a handheld homogenizer. After the addition of 200 μL of chloroform, the mixture was vortexed for 30 s and centrifuged at 10,000 rpm for 15 min at 4°C. The supernatant was collected, and 500 μL of isopropanol was introduced. The sample was centrifuged again at 10,000 rpm for 10 min at 4°C, and the lower phase was discarded. Subsequently, 75% ethanol was added, and the sample was centrifuged at 7,000 rpm for 5 min at 4°C. The ethanol was removed, and the RNA was dissolved in RNA-free water for concentration determination. Complementary DNA was synthesized using a reverse transcription kit (TianGen Biotech, Beijing, China). A 20 μL reaction mixture was prepared with the primers listed in Table 1. The PCR conditions were as follows: initial denaturation at 95°C, followed by 45 cycles of 95°C for 15 s, 60°C for 20 s, and 72°C for 35 s.

**Table 1:** Primer sequence of the genes

Genes	Primer	Sequence(5’-3’)
SREBP-1c	Sense	5’-AAGCAAATCACTGAAGGACCTGG-3’
	Anti-sense	5’-AAAGACAAGGGGCTACTCTGGGAG-3’
FAS	Sense	5’-AGGGGTCGACCTGGTCCTCA-3’
	Anti-sense	5’-GCCATGCCCAGAGGGTGGTT-3’
ACOX1	Sense	5’-TATTCGGCTATGACTGGGCACA-3’
	Anti-sense	5’-GATGGATACTTTCTCGGCAGGA-3’
PPAR-α	Sense	5’-GGATGTCACACAATGCAATTCGCT-3’
	Anti-sense	5’-TCACAGAACGGCTTCCTCAGGTT-3’
TNF-α	Sense	5’-ATGGCCCAGACCCTCACA-3’
	Anti-sense	5’-TTGCTACGACGTGGGCTACA-3’
IL-6	Sense	5’-GCTTAATTACACATGTTCTCTGGGAAA-3’
	Anti-sense	5’-CAAGTGCATCATCGTTGTTCATAC-3’
IL-1	Sense	5’-GACCTTCCAGGATGAGGACA-3’
	Anti-sense	5’-AGCTCATATGGGTCCGACAG-3’
β-Activin	Sense	5’-AGCCTTCCTTCTTGGGTATGG-3’
	Anti-sense	5’-CACTTGCGGTGCACGATGGAG-3’

### Statistical analysis

All data are presented as means ± standard deviations. Statistical analysis was performed using SPSS (version 23.0; IBM Corp., Armonk, NY, USA). Duncan’s multiple range test (*P* < 0.05) was applied to identify significant differences between groups, with distinct letters (a, b, c) representing differences, where a > b > c.

## Results

### Effects of DAPs and postbiotics on body weight

**Fig. 1:**
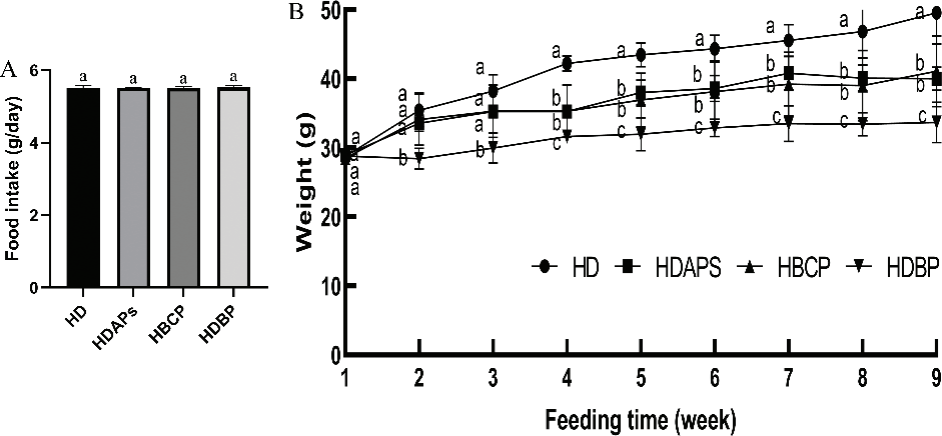
Effect of DAPs and postbiotics on body weight gain in mice. (A) Food intake and (B) body weight. HD, high-fat diet; HDAPs, high-fat diet + deer antler polysaccharides; HBCP, high-fat diet + *Bacillus coagulans* postbiotics; HDBP, high-fat diet + deer antler polysaccharides + *B. coagulans* postbiotics. Duncan’s multiple range test (*P* < 0.05): a > b > c.

Food intake among the mice in each group did not differ throughout the experiment (Fig. 1A). In the first 3 weeks, body weight did not differ among the HD, HDAPs, and HBCP groups, but the body weight of the HDBP group was significantly lower than those of the other three groups. Starting from the fourth week, the body weights of the HDAPs, HBCP, and HDBP groups decreased significantly compared to the HD group; the HDBP group had the most significant decrease (Fig. 1B).

At 9 weeks, the weight in the HD group was significantly higher than that at the start. Furthermore, the HDBP group had the most significant decrease in weight compared to the HD group (Fig. 1B). Thus, the combined use of DAPs and postbiotics was more effective than DAPs or *B. coagulans* postbiotics alone.

### Effects of DAPs and postbiotics on blood parameters

**Fig. 2:**
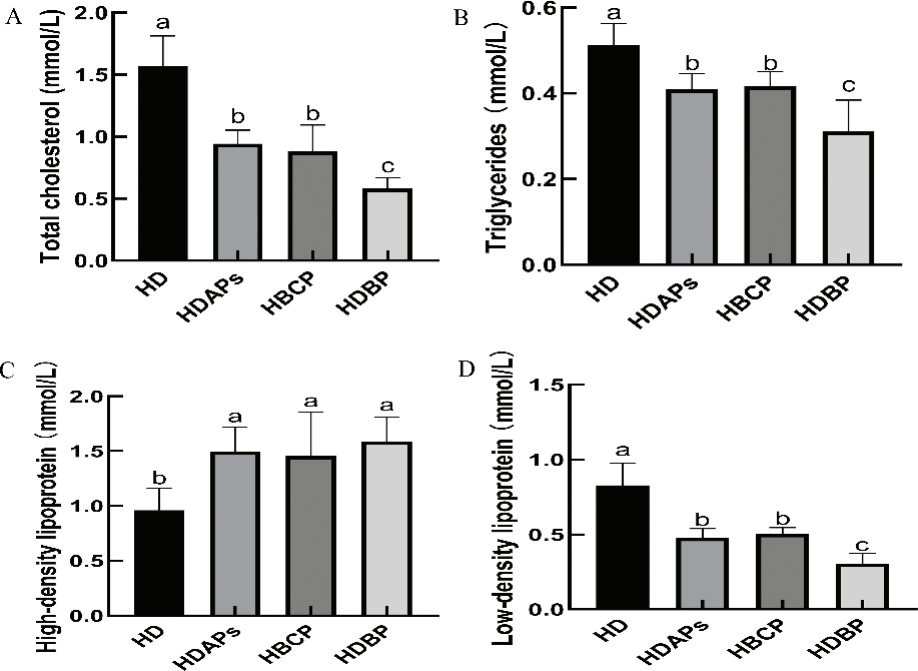
Effects of DAPs and postbiotics on blood parameters of mice. (A) Level of total cholesterol, (B) level of triglyceride, (C) level of high-density lipoprotein, and (D) level of low-density lipoprotein. HD, high-fat diet; HDAPs, high-fat diet + deer antler polysaccharides; HBCP, high-fat diet + *Bacillus coagulans* postbiotics; HDBP, high-fat diet + deer antler polysaccharides + *B. coagulans* postbiotics. Duncan’s multiple range test (*P* < 0.05): a > b > c.

The levels of TC, TG, and LDL-C were significantly elevated, while the level of HDL-C was notably reduced in the HD group compared to the other three groups. This observation suggests that lipid metabolism disorders were present in mice (Fig. 2). In contrast, the HDBP group exhibited marked improvements, with significantly reduced levels of TC, TG, and LDL-C, and a substantial increase in HDL-C compared to the HD group.

### Effects of DAPs and postbiotics on liver and adipose tissue

**Fig. 3:**
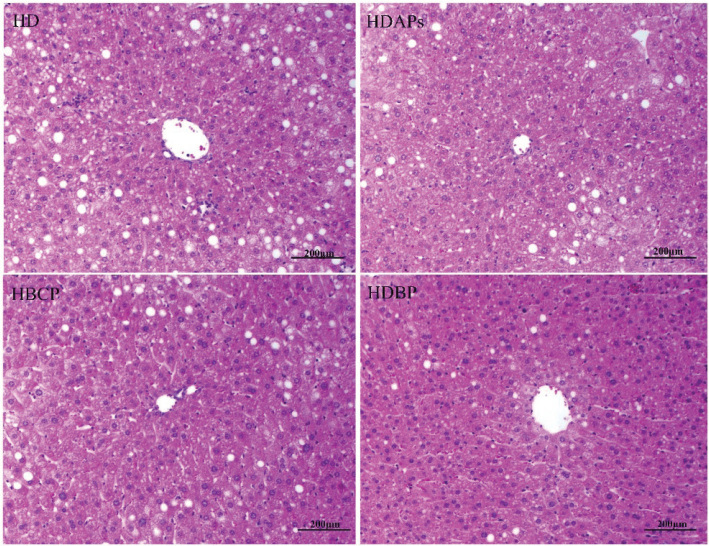
Effect of DAPs and postbiotics on liver lipid droplet accumulation in mice. HD, high-fat diet; HDAPs, high-fat diet + deer antler polysaccharides; HBCP, high-fat diet + *Bacillus coagulans* postbiotics; HDBP, high-fat diet + deer antler polysaccharides + *B. coagulans* postbiotics.

Considerable lipid droplet formation occurred in the liver tissue of mice from the HD group, whereas lipid droplet formation was markedly reduced in the HDBP group (Fig. 3). These results indicate that the combined use of DAPs and postbiotics inhibited lipid synthesis, reduced fat accumulation, and prevented abnormal enlargement of the liver. Moreover, the combined supplementation produced more considerable effects than the individual use of DAPs or postbiotics.

**Fig. 4:**
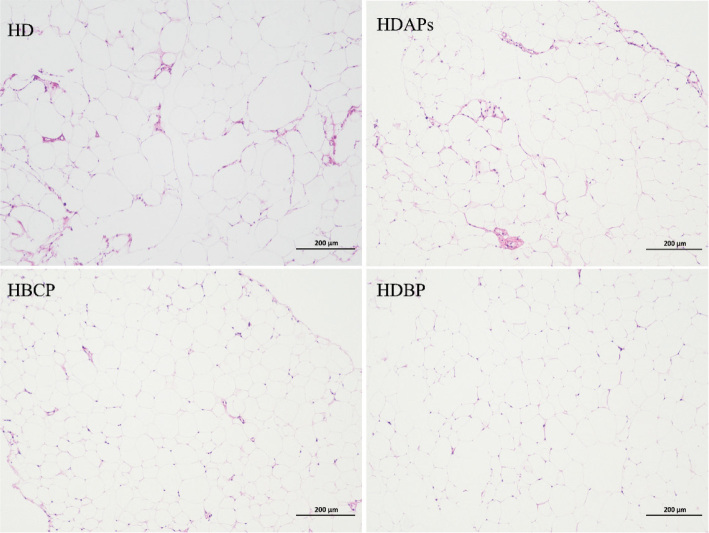
Effect of DAPs and postbiotics on adipocytes of mice. HD, high-fat diet; HDAPs, high-fat diet + deer antler polysaccharides; HBCP, high-fat diet + *Bacillus coagulans* postbiotics; HDBP, high-fat diet + deer antler polysaccharides + *B. coagulans* postbiotics.

In the HD group, adipocytes in the abdominal adipose tissue were larger than those in the other groups and unequally sized; enlarged lipid droplets with signs of cell fusion were also observed, suggesting a tendency for giant cell formation. In contrast, following the combined supplementation of DAPs and postbiotics, the adipocytes in the HDBP group were considerably smaller and more uniformly sized than those in the HD group (Fig. 4).

### Effects of DAPs and postbiotics on lipogenic and inflammatory gene expression

**Fig. 5:**
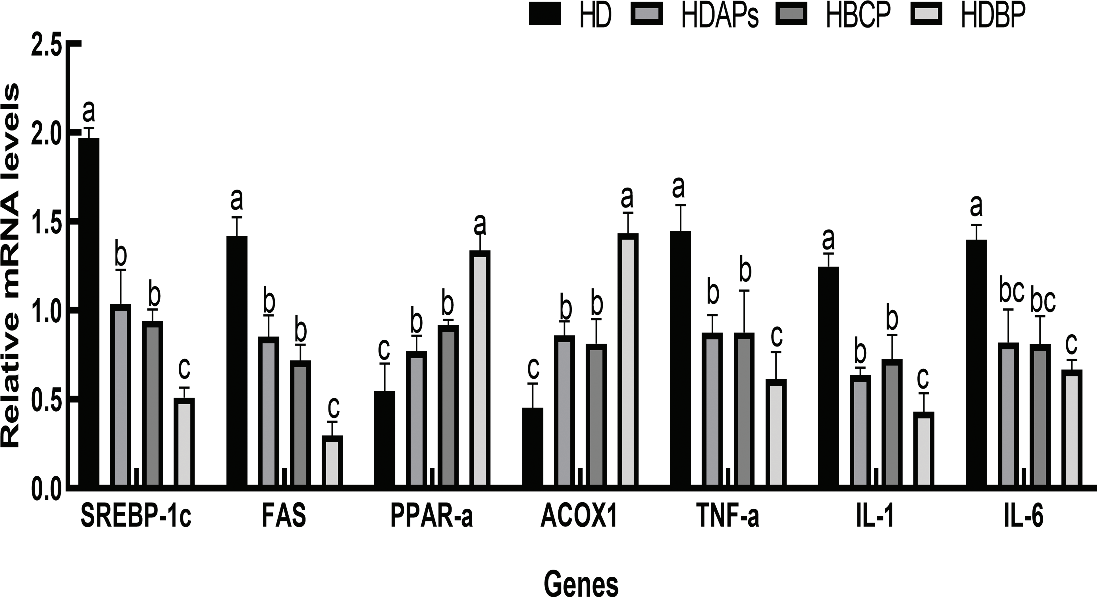
Effect of DAPs and postbiotics on gene expression of lipid production and inflammation in mice. HD, high-fat diet; HDAPs, high-fat diet + deer antler polysaccharides; HBCP, high-fat diet + *Bacillus coagulans* postbiotics; HDBP, high-fat diet + deer antler polysaccharides + *B. coagulans* postbiotics. Duncan’s multiple range test (*P* < 0.05): a > b > c.

The expression levels of SREBP-1c and FAS were significantly reduced, whereas those of PPAR-α and ACOX1 were markedly increased in the HDBP group compared to the HD group. Furthermore, the expressions of inflammation-related genes, including TNF-α, IL-6, and IL-1, were significantly higher in the HDBP group than in the HD group. These results suggest that the combined supplementation of DAPs and postbiotics may have a beneficial effect on regulating both lipid metabolism and the expression of inflammation-related genes (Fig. 5).

## Discussion

In this study, combined supplementation with DAPs and postbiotics effectively modulated serum lipid parameters. DAPs may enhance lipid metabolism by improving hepatic function and promoting the expression of genes involved in fatty acid oxidation, thereby reducing lipid accumulation (35). Postbiotics can influence gut microbiota composition, improving intestinal barrier function and reducing systemic inflammation, which can lower cholesterol absorption and promote bile acid synthesis (36). The synergistic effect of these two components may create a favorable environment for lipid homeostasis and metabolic health. Previous studies have demonstrated that *B. coagulans* postbiotics elicit good regulatory effects on elevated serum lipid levels and liver steatosis (37). Our results are consistent with this, as we found that combined supplementation with DAPs and postbiotics significantly affected the regulation of serum lipids. In particular, the HDBP group exhibited significantly reduced levels of TC, TG, and LDL-C, along with a notable increase in HDL-C compared to the HD group. This combined supplementation method shows promises for regulating lipid profiles, potentially providing a novel approach to managing obesity and associated metabolic disorders.

*SREBP-1c* is an important regulatory gene for lipogenesis as its activation increases *FAS* expression, resulting in lipid accumulation (38, 39). PPAR-α is an important member of the nuclear receptor super transcription factor family that activates ACOX1, promoting fat oxidation in the body (40). Significantly increased *PPAR-α* and *ACOX1* expression suggests enhanced fatty acid oxidation. PPAR-α plays a crucial role in lipid catabolism and energy expenditure, promoting the uptake and oxidation of fatty acids in the liver and muscle (41). *ACOX1* upregulation, an enzyme involved in the β-oxidation of fatty acids, further supports this notion. We observed decreased *SREBP-1c* and *FAS* levels in the HDBP group compared to the HD group, suggesting an inhibitory effect on lipogenesis. SREBP-1c is a critical transcription factor that regulates gene expression in fatty acid and triglyceride synthesis (10, 42). Reduced *SREBP-1c* expression implies that combined supplementation may hinder excessive fat accumulation by downregulating lipogenic pathways (43). In contrast, *PPAR-α* and *ACOX1* expressions increased significantly. Collectively, these findings suggest that DAPs and postbiotics act synergistically to promote lipid oxidation and limit lipid accumulation, counteracting obesity.

Additionally, we observed a significant decrease in the expression of inflammation-related genes, such as *TNF-α*, *IL-6*, and *IL-1*, in the HDBP group compared to the HD group. Obesity is a chronic disease accompanied by inflammation, which in turn increases the risk of diseases such as diabetes and cancer (44). This finding is intriguing, as increased expression of these pro-inflammatory cytokines typically correlates with obesity and metabolic disorders. However, the role of inflammation in metabolic regulation is complex and context dependent (45, 46). Chronic low-grade inflammation is a hallmark of obesity, contributing to insulin resistance and metabolic dysregulation (47). Reduced expression of *TNF-α* and *IL-6* may reflect an adaptive response to improve inflammatory signaling pathways that could enhance lipid mobilization and energy expenditure. This result suggests that combining DAPs and postbiotics might promote a balanced inflammatory response that supports metabolic health while also facilitating weight loss (48, 49). While the therapeutic potential of DAPs and postbiotics has been widely discussed, their safety and toxicological properties are equally important in assessing their suitability for long-term use. Studies on DAPs have shown that they are safe when taken at appropriate doses, with no significant acute toxicity observed in animal models (50). In addition, long-term studies of DAPs supplementation have shown no major adverse effects, indicating a good safety profile. Similarly, postbiotics, which are byproducts of probiotic microorganisms, have also been found to have a generally good safety profile. Studies have shown that postbiotics, such as microbial cell wall fragments, metabolites, and other bioactive components, are well tolerated in animal and human models (51). While the safety of velvet antler polysaccharides and postbiotics is well supported, more research is needed to evaluate the safety of their long-term use, especially in vulnerable populations such as those with compromised immune systems, and this work will also be the focus of our future research.

The interplay between lipid metabolism and inflammation is critical in understanding obesity and its associated complications. The dual effect of downregulating lipogenic genes while upregulating fatty acid oxidation markers, coupled with an increase in inflammatory markers, suggests a complex regulatory network where inflammation may serve both protective and detrimental roles. Modifying these pathways through dietary interventions, like DAPs and postbiotics, highlights the potential of these compounds in obesity management. Such combinations could be explored further in clinical settings by assessing improvements in lipid metabolism and possibly the inflammatory response. This study provides new insights into the potential therapeutic benefits of combined supplementation of DAPs and postbiotics for regulating obesity in mice. By elucidating the synergistic effects of these compounds, the results suggest that DAP and postbiotics may be promising candidates for developing novel dietary strategies to combat obesity and its associated metabolic disorders. Furthermore, exploring the efficacy of these compounds in human populations provides a potential natural and safe alternative for the treatment of obesity.

## Conclusion

Combined supplementation with DAPs and postbiotics elicited regulatory effects in obese mice, reducing their body weight and improving serum TC, TG, HDL-C, and LDL-C levels. Supplementation also improved the accumulation of lipid droplets in the liver and fat hypertrophy. Finally, supplementation with DAPs and postbiotics significantly decreased *SREBP-1c* and *FAS* expression (lipid-forming genes), significantly increased *PPAR-α* and *ACOX1* expression, and significantly decreased *TNF-α*, *IL-6*, and *IL-1* expression (inflammatory genes). This study was conducted over 9 weeks, which may not fully capture the long-term effects of DAPs and postbiotics on obesity and metabolic health. Longer-term studies are needed to assess the sustainability of the observed benefits and potential risks of prolonged supplementation. While the study provides valuable insights in a mouse model, the results need to be validated in human clinical trials. Differences in physiology between mice and humans may limit the direct applicability of these findings to human obesity management. Although key lipid metabolism and inflammation-related genes were assessed, other important pathways and factors involved in obesity, such as gut microbiota composition, insulin signaling, and adipokine levels, were not investigated in this study.

## Data Availability

The data presented in this study are available upon request from the corresponding author.

## References

[1] Goossens GH. The metabolic phenotype in obesity: fat mass, body fat distribution, and adipose tissue function. Obes Facts 2017; 10(3): 207–15. doi: 10.1159/000471488 28564650 PMC5644968

[2] Hruby A, Hu FBJP. The epidemiology of obesity: a big picture. Pharmacoeconomics 2015; 33: 673–89. doi: 10.1007/s40273-014-0243-x 25471927 PMC4859313

[3] Schwartz MW, Seeley RJ, Zeltser LM, et al. Obesity pathogenesis: an endocrine society scientific statement. Endocrine reviews 2017; 38(4): 267–96. doi: 10.1210/er.2017-00111 28898979 PMC5546881

[4] Swinburn BA, Sacks G, Hall KD, et al. The global obesity pandemic: shaped by global drivers and local environments. The lancet 2011; 378(9793): 804–14. doi: 10.1016/S0140-6736(11)60813-1 21872749

[5] Rosen ED, Spiegelman BMJC. What we talk about when we talk about fat. Cell 2014; 156(1): 20–44. doi: 10.1016/j.cell.2013.12.012 24439368 PMC3934003

[6] Kim YJ, Park JW, Kim JW, et al. Computerized automated quantification of subcutaneous and visceral adipose tissue from computed tomography scans: development and validation study. JMIR medical informatics 2016; 4(1): e4923. doi: 10.2196/medinform.4923 PMC4759454 26846251

[7] Mlinar B, Marc J, Janež A, Pfeifer MJCca. Molecular mechanisms of insulin resistance and associated diseases. Clinica chimica acta 2007; 375(1–2): 20–35. doi: 10.1016/j.cca.2006.07.005 16956601

[8] Albuquerque D, Stice E, Rodriguez-Lopez R, Manco L, Nobrega C. Current review of genetics of human obesity: from molecular mechanisms to an evolutionary perspective. Mol Genet Genomics 2015; 290(4): 1191–221. doi: 10.1007/s00438-015-1015-9 25749980

[9] Barbagallo F, Condorelli RA, Mongioi LM, et al. Molecular mechanisms underlying the relationship between obesity and male infertility. Metabolites 2021; 11(12): 840. doi: 10.3390/metabo11120840 34940598 PMC8706114

[10] Kolehmainen M, Vidal H, Alhava E, Uusitupa MI. Sterol regulatory element binding protein 1c (SREBP-1c) expression in human obesity. Obes Res 2001; 9(11): 706–12. doi: 10.1038/oby.2001.95 11707537

[11] Dentin R, Girard J, Postic C. Carbohydrate responsive element binding protein (ChREBP) and sterol regulatory element binding protein-1c (SREBP-1c): two key regulators of glucose metabolism and lipid synthesis in liver. Biochimie 2005; 87(1): 81–6. doi: 10.1016/j.biochi.2004.11.008 15733741

[12] Tahri-Joutey M, Andreoletti P, Surapureddi S, Nasser B, Cherkaoui-Malki M, Latruffe N. Mechanisms mediating the regulation of peroxisomal fatty acid beta-oxidation by PPARalpha. Int J Mol Sci 2021; 22(16): 8969. doi: 10.3390/ijms22168969 34445672 PMC8396561

[13] Huang J, Viswakarma N, Yu S, et al. Progressive endoplasmic reticulum stress contributes to hepatocarcinogenesis in fatty acyl-CoA oxidase 1-deficient mice. Am J Pathol 2011; 179(2): 703–13. doi: 10.1016/j.ajpath.2011.04.030 21801867 PMC3157234

[14] Mohammad IJ, Kashanian S, Rafipour R, Aljwaid H, Hashemi S. Evaluation of the relationship of cytokines concentrations tumor necrosis factor-alpha, interleukin-6, and C-reactive protein in obese diabetics and obese non-diabetics: a comparative study. Biotechnol Appl Biochem 2024; 71(2): 272–9. doi: 10.1002/bab.2539 38054266

[15] Cooke AA, Connaughton RM, Lyons CL, McMorrow AM, Roche HM. Fatty acids and chronic low grade inflammation associated with obesity and the metabolic syndrome. Eur J Pharmacol 2016; 785: 207–14. doi: 10.1016/j.ejphar.2016.04.021 27083551

[16] Lee YH, Pratley RE. The evolving role of inflammation in obesity and the metabolic syndrome. Curr Diab Rep 2005; 5(1): 70–5. doi: 10.1007/s11892-005-0071-7 15663921

[17] Wing RR, Phelan SJTAjocn. Long-term weight loss maintenance. The American journal of clinical nutrition 2005; 82(1): 222S–5S. doi: 10.1093/ajcn.82.1.222S 16002825

[18] Foster-Schubert KE, Cummings DEJEr. Emerging therapeutic strategies for obesity. Endocrine reviews 2006; 27(7): 779–93. doi: 10.1210/er.2006-0041 17122357

[19] Chan Y, Ng SW, Tan JZX, et al. Natural products in the management of obesity: fundamental mechanisms and pharmacotherapy. South African Journal of Botany 2021; 143: 176–97. doi: 10.1016/j.sajb.2021.07.026

[20] Park S-J, Sharma A, Lee H-JJIJoMS. Postbiotics against obesity: perception and overview based on pre-clinical and clinical studies. International Journal of Molecular Sciences 2023; 24(7): 6414. doi: 10.3390/ijms24076414 37047387 PMC10095054

[21] Sun H, Xiao D, Liu W, et al. Well-known polypeptides of deer antler velvet with key actives: modern pharmacological advances. Naunyn Schmiedebergs Arch Pharmacol 2024; 397(1): 15–31. doi: 10.1007/s00210-023-02642-y 37555852

[22] Wu F, Li H, Jin L, et al. Deer antler base as a traditional Chinese medicine: a review of its traditional uses, chemistry and pharmacology. Journal of Ethnopharmacology 2013; 145(2): 403–15. doi: 10.1016/j.jep.2012.12.008 23246455

[23] Orassay A, Sadvokassova D, Berdigaliyev A, et al. Deer antler extract: pharmacology, rehabilitation and sports medicine applications. Pharmacological Research-Modern Chinese Medicine 2024; 10: 100316. doi: 10.1016/j.prmcm.2023.100316

[24] Sui Z, Zhang L, Huo Y, Zhang Y. Bioactive components of velvet antlers and their pharmacological properties. J Pharm Biomed Anal 2014; 87: 229–40. doi: 10.1016/j.jpba.2013.07.044 24029381

[25] Chang J-S, Lin H-J, Deng J-S, et al. Preventive effects of velvet antler (Cervus elaphus) against lipopolysaccharide-induced acute lung injury in mice by inhibiting MAPK/NF-κB activation and inducing AMPK/Nrf2 pathways. Evidence-Based Complementary and Alternative Medicine 2018; 2018(1): 2870503. doi: 10.1155/2018/2870503 29483931 PMC5816838

[26] Cheng W-J, Yang H-T, Chiang C-C, et al. Deer velvet antler extracts exert anti-inflammatory and anti-arthritic effects on human rheumatoid arthritis fibroblast-like synoviocytes and distinct mouse arthritis. The American Journal of Chinese Medicine 2022; 50(06): 1617–43. doi: 10.1142/S0192415X22500689 35850642

[27] Lei Z, Ji B-P, Bo L, et al. Immunomodulatory effects of aqueous extract of velvet antler (Cervus elaphus Linnaeus) and its simulated gastrointestinal digests on immune cells in vitro. Journal of Food and Drug Analysis 2009; 17(4): 1. doi: 10.38212/2224-6614.2595

[28] Cui XS, Kim HI, Cho SKJJoAS, Technology. Effect of the water soluble extracts from velvet antler on lipid metabolism and blood components in rats. Journal of Animal Science and Technology 2008; 50(3): 417–28.

[29] Ding Y, Wang Y, Jeon BT, Moon SH, Lee SH. Enzymatic hydrolysate from velvet antler suppresses adipogenesis in 3T3-L1 cells and attenuates obesity in high-fat diet-fed mice. EXCLI J 2017; 16: 328–39. doi: 10.17179/excli2016-638 28507477 PMC5427470

[30] Bourebaba Y, Marycz K, Mularczyk M, Bourebaba L. Postbiotics as potential new therapeutic agents for metabolic disorders management. Biomed Pharmacother 2022; 153: 113138. doi: 10.1016/j.biopha.2022.113138 35717780

[31] Li HY, Zhou DD, Gan RY, et al. Effects and mechanisms of probiotics, prebiotics, synbiotics, and postbiotics on metabolic diseases targeting gut microbiota: a narrative review. Nutrients 2021; 13(9): 3211[EB/OL]. doi: 10.3390/nu13093211 34579087 PMC8470858

[32] Napolitano M, Fasulo E, Ungaro F, et al. Gut dysbiosis in irritable bowel syndrome: a narrative review on correlation with disease subtypes and novel therapeutic implications. Microorganisms 2023; 11(10): 2369. doi: 10.3390/microorganisms11102369 37894027 PMC10609453

[33] Huang J, Jiang R, Wang Y. Effects of the probiotic Bacillus coagulans BC69 on the metabolic and histological alterations induced by a high-sugar and high-fat diet in C57BL/6J mice. Food Funct 2023; 14(14): 6596–609. doi: 10.1039/d3fo01104f 37395073

[34] Cutting SM. Bacillus probiotics. Food Microbiol 2011; 28(2): 214–20. doi: 10.1016/j.fm.2010.03.007 21315976

[35] Jiang N, Zhang S, Zhu J, Shang J, Gao X. Hypoglycemic, hypolipidemic and antioxidant effects of peptides from red deer antlers in streptozotocin-induced diabetic mice. Tohoku J Exp Med 2015; 236(1): 71–9. doi: 10.1620/tjem.236.71 25985857

[36] Cani PD, Bibiloni R, Knauf C, et al. Changes in gut microbiota control metabolic endotoxemia-induced inflammation in high-fat diet-induced obesity and diabetes in mice. Diabetes 2008; 57(6): 1470–81. doi: 10.2337/db07-1403 18305141

[37] Hsieh RH, Chien YJ, Lan WY, et al. Bacillus coagulans TCI711 supplementation improved nonalcoholic fatty liver by modulating gut microbiota: a randomized, placebo-controlled, clinical trial. Curr Dev Nutr 2024; 8(3): 102083. doi: 10.1016/j.cdnut.2024.102083 38510931 PMC10951533

[38] Chen H, Tan H, Wan J, et al. PPAR-gamma signaling in nonalcoholic fatty liver disease: pathogenesis and therapeutic targets. Pharmacol Ther 2023; 245: 108391. doi:10.1016/j.pharmthera.2023.108391 36963510

[39] Puengel T, Liu H, Guillot A, Heymann F, Tacke F, Peiseler M. Nuclear receptors linking metabolism, inflammation, and fibrosis in nonalcoholic fatty liver disease. Int J Mol Sci 2022; 23(5): 2668. doi: 10.3390/ijms23052668 35269812 PMC8910763

[40] Huo X, Yang S, Sun X, Meng X, Zhao Y. Protective effect of glycyrrhizic acid on alcoholic liver injury in rats by modulating lipid metabolism. Molecules 2018; 23(7): 1623. doi: 10.3390/molecules23071623 30857301 PMC6429446

[41] Coll T, Rodriguez-Calvo R, Barroso E, et al. Peroxisome proliferator-activated receptor (PPAR) beta/delta: a new potential therapeutic target for the treatment of metabolic syndrome. Curr Mol Pharmacol 2009; 2(1): 46–55. doi: 10.2174/1874467210902010046 20021445

[42] Ferre P, Foufelle F. Hepatic steatosis: a role for de novo lipogenesis and the transcription factor SREBP-1c. Diabetes Obes Metab 2010; 12 Suppl 2: 83–92. doi: 10.1111/j.1463-1326.2010.01275.x 21029304

[43] Illesca P, Valenzuela R, Espinosa A, et al. Hydroxytyrosol supplementation ameliorates the metabolic disturbances in white adipose tissue from mice fed a high-fat diet through recovery of transcription factors Nrf2, SREBP-1c, PPAR-gamma and NF-kappaB. Biomed Pharmacother 2019; 109: 2472–81. doi: 10.1016/j.biopha.2018.11.120 30551508

[44] Ying W, Fu W, Lee YS, Olefsky JM. The role of macrophages in obesity-associated islet inflammation and beta-cell abnormalities. Nat Rev Endocrinol 2020; 16(2): 81–90. doi: 10.1038/s41574-019-0286-3 31836875 PMC8315273

[45] Miller YI, Shyy JY. Context-dependent role of oxidized lipids and lipoproteins in inflammation. Trends Endocrinol Metab 2017; 28(2): 143–52. doi: 10.1016/j.tem.2016.11.002 27931771 PMC5253098

[46] Collins KH, Herzog W, MacDonald GZ, et al. Obesity, metabolic syndrome, and musculoskeletal disease: common inflammatory pathways suggest a central role for loss of muscle integrity. Front Physiol 2018; 9: 112. doi: 10.3389/fphys.2018.00112 29527173 PMC5829464

[47] Varra FN, Varras M, Varra VK, Theodosis-Nobelos P. Molecular and pathophysiological relationship between obesity and chronic inflammation in the manifestation of metabolic dysfunctions and their inflammation‑mediating treatment options (Review). Mol Med Rep 2024; 29(6): 95. doi: 10.3892/mmr.2024.13219 38606791 PMC11025031

[48] Dali-Youcef N, Mecili M, Ricci R, Andres E. Metabolic inflammation: connecting obesity and insulin resistance. Ann Med 2013; 45(3): 242–53. doi: 10.3109/07853890.2012.705015 22834949

[49] Bastard JP, Maachi M, Lagathu C, et al. Recent advances in the relationship between obesity, inflammation, and insulin resistance. Eur Cytokine Netw 2006; 17(1): 4–12. 16613757

[50] Yang H, Wang L, Sun H, He X, Zhang J, Liu FJJoFB. Anticancer activity in vitro and biological safety evaluation in vivo of Sika deer antler protein. Journal of Food Biochemistry 2017; 41(6): e12421. doi: 10.1111/jfbc.12421

[51] Ji J, Jin W, Liu SJ, Jiao Z, Li XJM. Probiotics, prebiotics, and postbiotics in health and disease. MedComm 2023; 4(6): e420. doi: 10.1002/mco2.420 37929014 PMC10625129

